# Effect of Rosin Modification on the Visual Characteristics of Round Bamboo Culm

**DOI:** 10.3390/polym13203500

**Published:** 2021-10-12

**Authors:** Na Su, Changhua Fang, Hui Zhou, Tong Tang, Shuqin Zhang, Xiaohuan Wang, Benhua Fei

**Affiliations:** 1 Department of Biomaterials, International Center for Bamboo and Rattan, Beijing 100102, China; yuhesu122216@126.com (N.S.); cfang@icbr.ac.cn (C.F.); zhouhui@icbr.ac.cn (H.Z.); zhangshuqin@icbr.ac.cn (S.Z.); 2 SFA and Beijing Co-Built Key Laboratory of Bamboo and Rattan Science & Technology, State Forestry Administration, Beijing 100102, China; 3 Environmental Design, Institute of Art & Design, Qilu University of Technology, Jinan 250353, China; Tangtong@icbr.ac.cn; 4 Beijing Forestry Machinery Institute of National Forestry and Grassland Administration, Beijing 100013, China

**Keywords:** rosin modification, round bamboo culm, visual characteristics, eye tracking

## Abstract

Rosin was used to treat round bamboo culm using the impregnation method. The quantitative color and gloss measurements combined with a qualitative eye tracking experiment were used to evaluate the effect of rosin treatment under different temperatures on the visual characteristics of the bamboo surface. Surface morphology analysis was also used to explore the mechanism of modification. The results showed that proper heating of the modified system was conducive to the formation of a continuous rosin film, which increased the gloss value. The maximum gloss value of 19.6 achieved at 50 °C was 122.7% higher than the gloss value of the control group. Heating decreased the brightness of the bamboo culm and changed the color from the green and yellow tones to red and blue. Additionally, at temperatures higher than 60 °C, the bamboo epidermal layer was damaged or shed, and stripes formed on the culm surface. The density of these stripes increased with an increase in treatment temperature. Eye movement experiment and subjective evaluation showed that high gloss would produce dazzling feeling, such as at 50 °C, while low gloss will appear dim, such as at 80 °C, while the gloss at 40 °C and 60 °C were appropriate. Additionally, the solid color surface below 60 °C had a large audience of about 73%, and the striped surface above 60 °C was preferred by 27% of the subjects.

## 1. Introduction

Since ancient times, people have used bamboo and wood to decorate the interior environment and to make indoor furniture [1,2]. Round bamboo in particular complements various natural materials in construction and interior design where it is used to create a traditional oriental style [3]. Round bamboo displays an impressive range of attractive properties, including a high ratio of weight to strength, easy workability, natural aesthetics, and environmental sustainability [1,4,5]. Among these, the visual characteristics (such as color, gloss and texture) play one of the most visual roles [4,5]. Visual characteristics not only affect the aesthetic of furniture or architecture, but also the psychological and physiological well-being of users by influencing psychology, communication, psychosomatic effects, visual ergonomics, etc. [6]. For example, the impression of a color and the message it conveys is of utmost importance in creating the psychological mood or ambiance that supports the function of a space [7,8]. Each color gives a different impression and conveys a specific meaning. With round bamboo culm, fresh bamboo is mostly green, while dried bamboo is mostly yellow [2,9]. Green induces a balancing, natural, and calm state, conveying a message in the interior space is of simplicity, security, and balance in the interior space [10]. Pastel yellow gives the impression of a sunny, friendly, and soft environment, with a message of stimulation, brightness, and coziness. Gloss also influences warmth with a high gloss, producing a stiff and cold impression [11]. Lastly, stripes or scars on the surface of the bamboo will also offer a simpler, more natural decorative effect [12]. 

The color related chemical components in bamboo mainly arise from lignin and some extract with unsaturated structure while the gloss may be more related to microstructure [13,14]. A smooth, low porosity surface will obtain a greater gloss but when the surface cells are exposed or damaged, diffuse reflection or absorption of light will reduce the gloss [15]. Therefore, application of treatment or finishing can change the color, gloss and even texture of the bamboo surface [16,17]. Feng et al., 2020 [18] reported that heat treatment above 200 °C will turn the bamboo brown, and indicated that this is due to the change in lignin structure or the oxidation of phenol compounds to quinones. Transparent finishing can also improve the gloss of bamboo and enhance the contrast of texture. Despite this, the visual quality is ignored in many processing treatments, which limits the practical applications of bamboo. 

**Figure 1:**
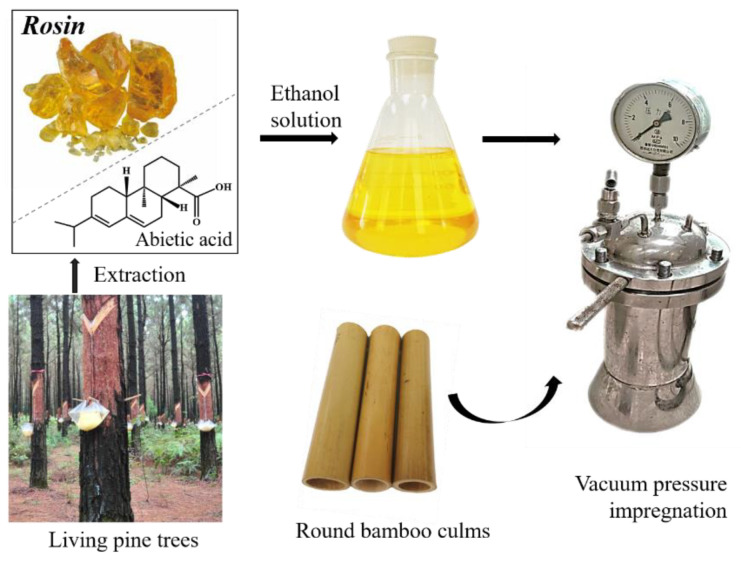
Schematic illustration of rosin impregnation of bamboo culms.

Rosin is a natural resin derived from living pine trees [19]. It is a common polymer monomer due to the presence of reactive groups, including carboxyl groups and conjugated double bonds in its molecules as shown in Figure 1 [20,21,22]. Rosin can produce polymerized rosin, maleated rosin and disproportionated rosin through addition reaction and polymerization, and can further synthesize polymer materials with different properties, which are widely used in glass fiber reinforced plastics and coatings [23,24]. In addition, rosin is often used in modifier or coating to improve the water resistance and visual properties because of its good hydrophobicity and gloss [25,26]. Over recent years, rosin and its derivatives have been used to modify nanocomposites, wood materials, wood-based panels, and packaging [27,28,29,30]. Dahlen et al. (2008) [31] reported that only 3% rosin could significantly increase the water resistance of wood. Dong et al. (2016) [29] used rosin to treat fast-growing poplar wood under vacuum pressure impregnation and showed that the wood density increased from 0.34 to 0.44 g/cm^3^ after the treatment, and the anti-swelling efficiency achieved 36.00%. Rosin treatment was used as a hydrophobic method for bamboo in our previous studies [25,32]. This treatment resulted in good hydrophobicity and improved the dimensional stability of round bamboo. Microtopography also showed that rosin formed a transparent film on the surface of the round bamboo culm. Since rosin exists as a light yellow transparent solid, as shown in Figure 1, rosin treatment of bamboo may change the visual characteristics of bamboo culm. However, this has not been studied so far.

Visual characteristics could be described by physical quantities and psychological quantities. The former can be described using physical parameters such as color, gloss and texture, while the latter consists of human visually induced psychological assessment. At present, measurements of color and gloss are conducted using colorimeters and glossmeters to obtain quantitative data, respectively. Visual quantitative psychological parameters can be obtained using eye tracking tests [33]. Eye movement technology is often used in human-computer interaction research, psychological exploration, on web pages and in graphic design evaluation [34,35,36,37]. It is based on the characteristics of directness, naturalness and bi-directionality of human line of sight [38]. The basic working principle of eye tracking is to use image processing technology to continuously record both the change in view with the infrared rays in the camera and the pupil reflex with a tracking camera which focuses on the eye [39]. The images are used to clarify the response behavior of the visual system to different objects by analyzing the trajectory of eye movement characteristics, such as saccades, gaze time and pupil switching. In recent years, this technology has been gradually used in the fields of furniture and architectural design [40,41].

In this study, to investigate the effect of natural resin rosin modification on the surface visual effect of round bamboo culm, the color and gloss of bamboo culm before and after rosin modification were quantitatively determined. Meanwhile, the eye tracking technology was used to evaluate the users’ preference for the visual characteristics of the bamboo culm surface. The combination of quantitative index and qualitative preference analysis to evaluate the visual effect of bamboo culm was used in this study to improve the scientificity and systematicness of the evaluation. In addition, to clarify the mechanism of color change, the surface microscopic morphology and chemical group of the bamboo culms were explored.

## 2. Materials and Methods

### 2.1. Impregnation of the Bamboo Culm with Rosin

The sampling of bamboo culm and preparation of rosin ethanol solution was consistent with our previous study [25,32]. Five-year-old Hong bamboo (*Phyllostachys iridencens* C.Y. Yao and S.Y. Chen), treated with steam at 130 °C for 1 h, were obtained from Hangzhou Suo Bamboo Industry Co., Ltd. (Suo Bamboo Industry Co., Ltd., Hangzhou, China). Culm internodes were selected for this study (as shown in Figure 1) from 1.5 to 4.0 m aboveground. The rosin was purchased from Jitian Chemical Co., Ltd. (Jitian Chemical Co., Ltd., Shenzhen, China). Rosin ethanol solutions with concentrations of 20 wt% were prepared (Figure 1). The rosin ethanol solutions were then used to impregnate bamboo culm with vacuum pressure impregnation, as outlined in our previous study [25,32]. However, different temperatures were used in the impregnation process to study the effect of temperature on the visual characteristics of bamboo culm. The temperature parameters are set to 25 °C (room temperature), 40 °C, 50 °C, 60 °C, 70 °C, and 80 °C. For all tests, eight replicates were performed.

### 2.2. Measurement of Color and Gloss 

A surface gloss test was conducted according to ISO (2014) [42]. The gloss of bamboo culm samples was measured using a WGG 60 glossmeter (Shanghai Precision Scientific Instrument Co., Ltd., Shanghai, China) at six points on the bamboo surface before and after rosin treatment. The chosen geometry was an incidence angle of 60° and results were based on a specular gloss value of 100.

A color test was performed using color space CIE L*a*b* [43]. The color changes of bamboo culms before and after rosin treatment were determined using an SP60 spectrophotometer (CC-6834, BYK, Grazrid, Germany) with a D65 light (standard light source) and a 10° view angle. Color changes were assessed at six points on the bamboo surface before and after rosin treatment. The CIE L*a*b* system consists of three perpendicular axes to describe color. The L* axis represents lightness, the a* axis represents the red-green factor, and the b* axis represents the yellow-blue factor. The overall color change (ΔE*) was determined as follows:

(1) $$\Delta E^{*}={\left[{\left(\Delta a^{*}\right)}^{2}+{\left(\Delta b^{*}\right)}^{2}+{\left(\Delta L^{*}\right)}^{2}\right]}^{1/2}$$

where Δa*, Δb*, and ΔL* represent the changes in a*, b* and L* between rosin-treated and control groups. 

### 2.3. Micromorphology Characteristics

The surface micromorphology of bamboo culm before and after rosin treatment at different temperatures was characterized using a field emission scanning electron microscope (FE-SEM) (XL30, FEI, Hillsboro, OR, USA). To highlight the distribution of rosin on the bamboo surface, the multi-Otsu thresholding algorithm was used to obtain the thresholds of different surface components using FIJI/Image J software.

### 2.4. Micromorphology Characteristics

Fourier transform infrared (FTIR) spectroscopy of rosin and rosin-treated bamboo culm at different temperature was conducted with a standard FTIR spectroscope (Perkin Elmer Inc., Shelton, CT, USA). The spectra from 4000 to 500 cm^−1^ were recorded at a 4-cm^−1^ resolution across 64 scans. 

### 2.5. Eye Tracking Test

In conjunction with the ClearView 2.7.0 software system, the Tobii X120 Eye-Tracker (Tobii X120, Tobii Technology AB, Danderyd, Sweden) was used to record the eye tracks of participants. This tracker consists of an observation host, computer and camera. Twenty-six volunteers (all students) were recruited from the Qilu University of Technology, including 25 males and 25 females. The health condition was tested, and all volunteers tested normal i.e., without any symptom of color blindness or weakness. The naked or corrected visual acuity for all volunteers was >1.0.

All bamboo culms (before and after rosin treatment) were placed about 600 mm away from the screen to follow the eye calibration procedure. The experimental aim was to explore the effect of different treatment temperatures on the visual effect of the bamboo culm surface. The total gaze time, gaze numbers, average gaze time and combined heat map (measured for each bamboo culm) were used as the eye-tracking indicators. 

Meanwhile, subjective reviews were completed right after watching, including color comfort, gloss comfort, texture preference, and texture density comfort. Among these, the color comfort and texture density comfort levels were discomfort, general, and comfort; the gloss comfort level was dim, moderate and dazzling; texture preferences were solid color and texture. The proportion of each grade was then counted.

## 3. Results and Discussion

### 3.1. Color and Gloss

**Figure 2:**
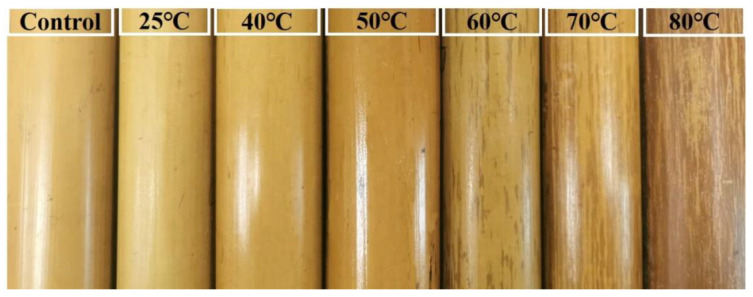
Effect of rosin treatment with different temperature on the color and gloss of bamboo culm.

As shown in Figure 2, the bamboo color varied from yellow to dark yellow to light brown with some stripes presenting as the temperature of the rosin treatment increased. Specifically, when the temperature was between 25–50 °C, the bamboo surface presented a solid color, and the color change was minimal. At temperatures ranging between 60–80 °C, brown stripes appeared on the bamboo surface. The surface of the control group presented as yellow with a certain gloss. The 25 °C and 40 °C treatment hardly changed the color of bamboo culm. However, the treatment at room temperature (25 °C) decreased the gloss value, while treatment at 40 °C increased the gloss value. The gloss of the rosin-treated bamboo culm was mainly a result of the rosin film, because the rosin itself is light yellow and transparent. The 25 °C treatment did not promote the formation of a continuous film of rosin. With additional heating (40 °C and 50 °C), the gloss value, which indicated that heating could promote the formation of a continuous film of rosin. However, when the temperature was higher than 60 °C, brown stripes appeared on the surface of bamboo, and the gloss decreased gradually. Moreover, stripe formation increased with the increase of treatment temperature, and even patches were formed at 80 °C.

**Figure 3:**
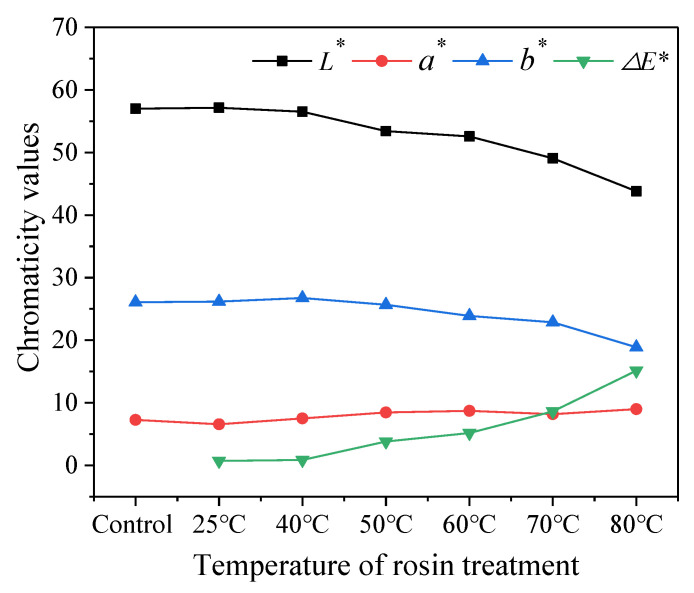
Chromaticity values of bamboo culm before and after the rosin treatment with different temperature.

The quantified chromaticity values are expressed in Table 1, while its trends are visually displayed in Figure 3. Firstly, the values of L*, a*, and b* showed little change at 25 °C, which indicated that the rosin treatment at room temperature had no obvious effect on the color of bamboo culm. This is consistent with images displayed in Figure 2. The L* value decreased with the increase in temperature, which indicated a decrease in brightness of the bamboo culm as treatment temperature increased. The a* value initially decreased but then increased gradually with a rising temperature, whereas the b* value decreased gradually with an increase in temperature (Table 1). These were indicative of color changes from the green and yellow zones to the red and blue zones. Feng, et al. found a similar phenomenon when investigating hygrothermal treatment on Moso bamboo [18]. Lastly, the change of ΔE* shows that an increase in temperature will increase the color difference. This is also seen in Figure 1. The chemical components related to color in bamboo mainly come from lignin and extract, because the structure of lignin and extract contains a large number of aromatic compounds, chromogenic groups and color assisting groups [44]. In this study, the structure of lignin and extract may have remained unchanged because the treatment temperature was not high enough for such a change. The change in color may be relative to the addition reaction of the carboxyl group in rosin or the oxidative discoloration of rosin itself. Rosin contains conjugated double bonds and carboxyl groups, which are prone to addition reactions, especially oxidation and discoloration, at an elevated temperature [45]. 

Additionally, the gloss value in Table 1 shows that rosin treatment improved the gloss of bamboo culm at all temperatures, except at room temperature (25 °C) and 80 °C. In detail, the gloss decreased by 3.2 at 25 °C when compared to the control group (8.8). With additional heating, the gloss increased perceptibly. At 50 °C, the gloss reached the highest value of 19.6, which was 122.7% higher than that of the control group. With the continuous increase in temperature, the gloss showed a downward trend. The gloss value of the bamboo culm was just 6.8 at 80 °C, which was lower than the control group.

### 3.2. Surface Topography Characteristics

**Figure 4:**
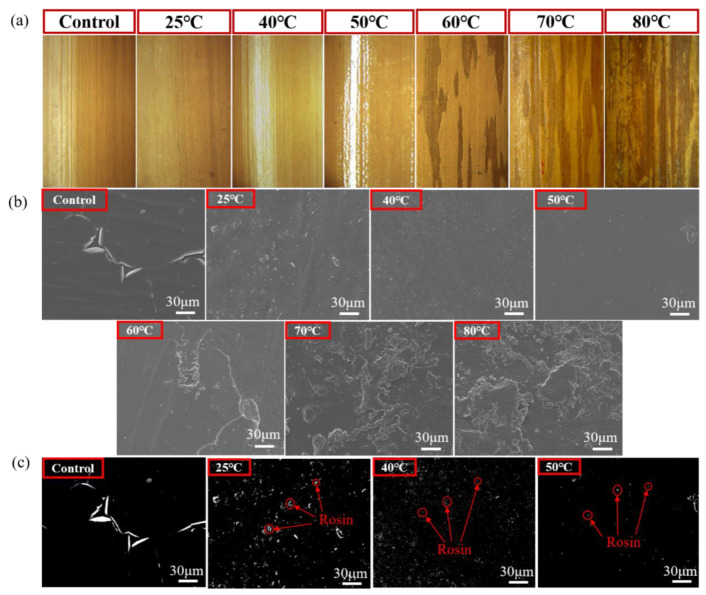
(**a**) Surface texture of bamboo culm observed using stereomicroscopy; (**b**) the surface micromorphology of bamboo culm observed using FE-SEM; (**c**) the surface micromorphology after thresholding by FIJI/Image J software.

The Figure 4a shows the surface morphology of bamboo culm observed using a stereomicroscope to clearly examine the surface texture and the changes before and after rosin modification under different temperatures. Firstly, when the treatment temperature is lower than 60 °C, the bamboo surface is relatively smooth with a uniform color. In detail, the surface of bamboo treated at room temperature (25 °C) is rough with white granular bulges while the surfaces of rosin-treated bamboo at 40 and 50 °C are smooth and glossy. At 60 °C, 70 °C, and 80 °C, clear brown stripes appear on the bamboo surface. In Figure 4a it is observed that the brown stripes are concave, which indicates that stripes are formed by the shedding of the bamboo epidermis during rosin treatment. Comparing 60 °C, 70 °C and 80 °C, it is observed that more of the bamboo epidermis is shed with the increase in treatment temperature. 

Figure 4b shows the surface micromorphology of bamboo culm using SEM. There is slight tearing in the epidermis of the control group. For bamboo culm treated at room temperature (25 °C), there are some granular bulges on the surface. Conversely, there are irregular depressions on the bamboo surface when treated at 40 °C. However, the surface of bamboo treated at 50 °C is very smooth. In order to more intuitively compare the surface morphology, images were threshold segmented using FIJI/Image J software, as shown in Figure 4c. In the thresholding images, the black represents the outline of the bamboo surface, while the white represents the outline of another substance, in this case rosin (Figure 4c). For the control group, the outline of the broken epidermis is significant. At 25 °C, 40 °C and 50 °C, the white dots are particles formed by rosin curing on the culm surface. It is evident that the particle size and the number both decrease with the increase in temperature as shown in Figure 4c. Overall, it is apparent that rosin forms the most continuous film at 50 °C, followed by 40 °C and 25 °C in succession. This may be related to the solubility of rosin at different temperatures. At room temperature, the particle size of the rosin ethanol system is larger than it is at 40 °C and 50 °C. Heating up the system can effectively reduce the particle size of rosin, which is conducive to the film-forming process. However, when the temperature reaches 60 °C, the bamboo epidermis is damaged to varying degrees. This may be a synergistic result of temperature, ethanol and rosin. In detail, there are a few but large depressions on the bamboo surface at 60 °C, while those depressions are more obvious and aggregated at 70 and 80 °C (Figure 4).

The bamboo surface morphology (Figure 4) also explains the change in gloss value. Between 25–50 °C, the gloss is mainly determined by the distribution of rosin on the surface where the continuity of rosin film improves with the decrease in rosin particle size. The SEM results also show that the surface roughness of bamboo decreased at these temperatures. Since the gloss increases with the decrease of roughness, the gloss of bamboo culm increases from 25 °C to 50 °C as shown in Table 1. Between 60–80 °C, the gloss of the bamboo culm is related not only to the continuity of rosin film, but also to the destruction and abscission of the epidermal layer. The epidermal layer of bamboo is composed of long cells, embolic cells, siliceous cells and stomata [46]. Long cells account for most of the area and are arranged in parallel along the grain while embolic and siliceous cells are short and inserted between the ranks of long cells. The epidermal layer is closely arranged without gaps, interspersed only with stomata. However, the subcutaneous layer and cortical structure beneath the epidermal layer are loose. According to the basic principles of optics [47], the higher the degree of reflection of bamboo to incident light, the higher the gloss value. For a loose or rough surface, incident light will form multiple reflections on the bamboo surface, increasing the probability of absorption, and producing a low gloss value. The breaking or exfoliation of the bamboo epidermal layer after rosin treatment at 60–80 °C would decrease the gloss of the bamboo culm due to the exposure of the loosely structured subcutaneous layer. Similarly, for control group, the tearing of the epidermal layer also produces a low gloss. 

### 3.3. FTIR Spectroscopy

**Figure 5:**
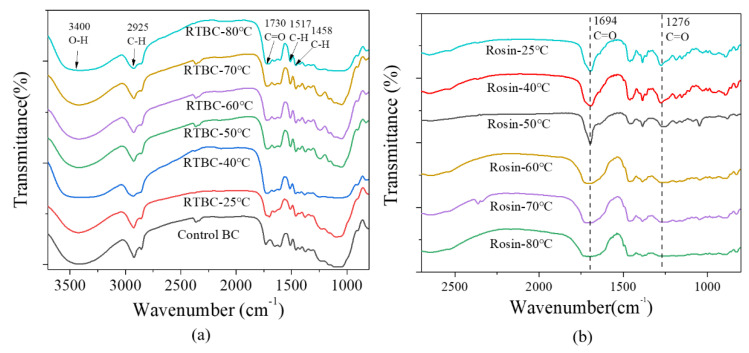
Fourier transform infrared spectroscopy spectra of (**a**) control bamboo culm and rosin-treated bamboo culm (RTBC) at different temperature; and (**b**) rosin at different temperature.

The FTIR spectrum of surface materials of bamboo culm and rosin at different temperatures were obtained to determine the reasons of discoloration of bamboo surface. The FTIR spectrum results are shown in Figure 5. First, the FTIR spectrum of bamboo surface materials shows the typical transmittance peaks of bamboo, e.g., 3400, 2925, 1730, 1517 and 1458 cm^−1^ [48,49]. The peaks at 3400 cm^−1^ and 1730 cm^−1^ correspond to O–H and C=O stretching vibration, while the peaks at 2925, 1517 and 1458 cm^−1^ correspond to the C–H stretching vibration. However, compared to the control bamboo, these typical transmittance peaks hardly changed after rosin treatment at different temperatures. Therefore, the discoloration of bamboo surface may have little relationship with bamboo surface chemicals. Additionally, the Figure 5b shows the typical transmittance peaks of rosin. Among these, the peaks at 1694 cm^−1^ and 1276 cm^−1^ corresponds to C=O stretching vibration, which belong to carbonyl group in carboxyl group [22,32]. Carboxyl group is a chromogenic group, which is easy to be oxidized, resulting in discoloration [50]. Compared the rosin at different temperatures, it can be seen that the peak intensity at 1694 cm^−1^ and 1276 cm^−1^ decreased and broadened with the increase of temperature. This means that the stretching vibration of carbonyl group is weakened, which may be due to the oxidation of carboxyl group of rosin under the action of heat. In detail, when the temperature rises to 50 °C, both of peak’s width at 1694 cm^−1^ and 1276 cm^−1^ gradually increase and the intensity gradually decrease. These are consistent with the discoloration of bamboo in Figure 2. The FTIR results also revealed that the discoloration of bamboo surface is caused by the oxidation discoloration of rosin under heating, rather than the material change of bamboo itself.

### 3.4. Eye Tracking Analysis

**Figure 6:**
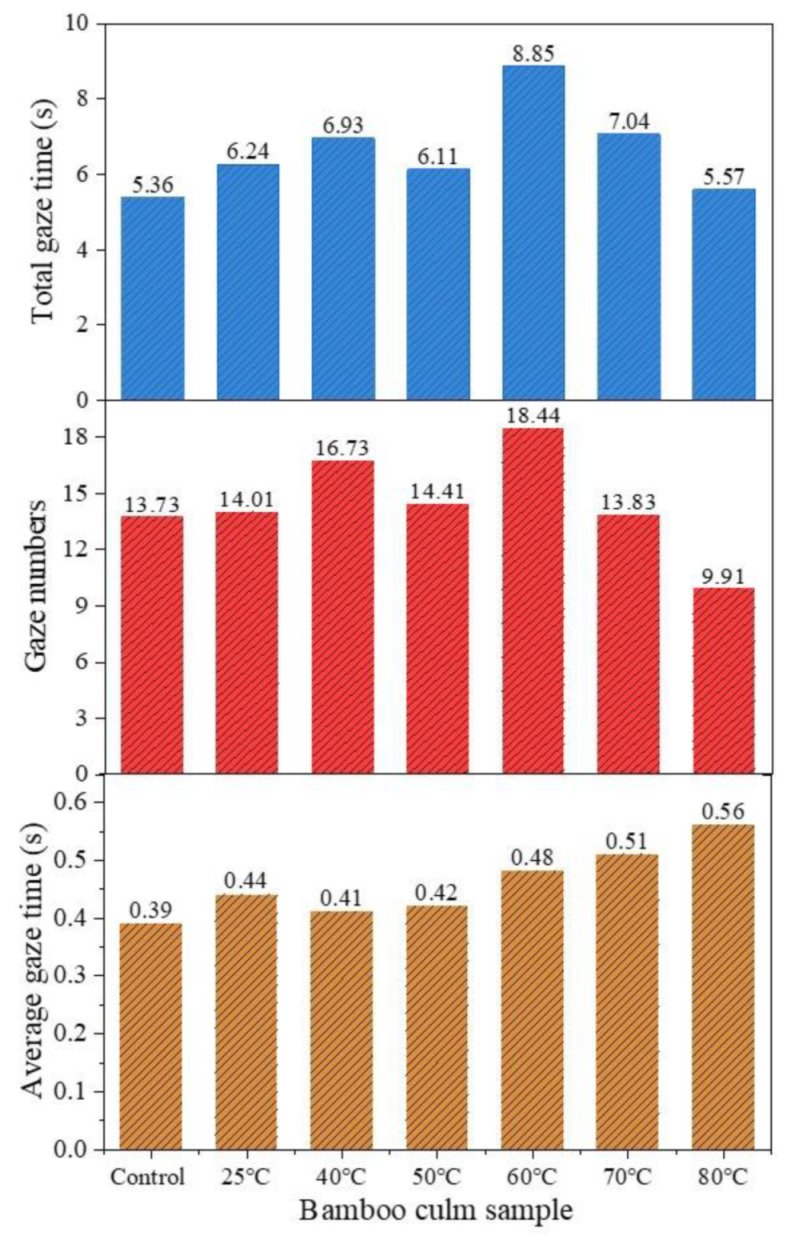
The total gaze time, gaze numbers, and average gaze time of bamboo culms.

Figure 6 presents the total gaze time, gaze numbers, and average gaze time before and after rosin treatment at different temperatures. The total gaze time represents the total time each subject looked at a particular culm during the observation period while gaze numbers refer to the number of times subjects looked at a specific culm. The average gaze time is the average of the duration of the subject’s line of sight on the bamboo culm each time. The gaze time and numbers reflect the volunteers’ attention to the bamboo culms [38]. The average gaze time is used to measure the difficulty of information extraction [37,38]. The larger the value, the more complex the subject and its details, and the longer the interpretation time. First, it can be seen that the gaze time of rosin-treated bamboo culms are all higher than that of the control. This suggests that the rosin treatment has increased the attention given to the bamboo culms by participants. The gaze time of the 60 °C treatment was the highest. Combined with surface color and texture, the brown stripes may have been the key factor attracting the subject’s attention. Research shows that certain knots on the wood surface will result in a certain decorative effect and give people a simple and natural feeling [51]. A similar phenomenon may have occurred with the bamboo surface. Additionally, the intensity of stripes may also affect feelings. Comparing the gaze time of bamboo culms treated at 60 °C, 70 °C and 80 °C, it is evident that with the increase of stripe density, the gaze time decreases, i.e., the degree of interest decreases. Furthermore, the lightness of the bamboo culm gradually decreases from 60 °C to 80 °C, suggesting that lightness may also influence people’s interest. This is also reflected in the bamboo culm treated at 50 °C. This culm had the lowest gaze time (Figure 6) despite having the smoothest surface (Figure 4b,c) and highest gloss value (19.6—Table 1) among all groups. This may be related to the lower lightness of the bamboo. Moreover, a high gloss has been shown to induce dizziness [52]. This may also be the reason for the reduction of gaze time for 50 °C. The gaze numbers show a similar trend to the total gaze time, except at 80 °C. The gaze number for 80 °C is lower than that of the control group, which suggests that the bamboo culm treated at 80 °C has no better visual effect than the control culm. The gaze time of 80 °C is also only slightly higher than the control. Additionally, the average gaze times of the treated bamboo culm are all higher than the control group and are positively correlated with temperature. The culms treated at 60–80 °C have longer average gaze times (0.48 s, 0.51 s and 0.56 s) than those treated at 25–50 °C (0.44 s, 0.41 s, and 0.42 s) (Figure 6). This may be due to the brown stripes of the culms treated at higher temperatures which provide more complexity than the uniform surface of those treated at lower temperatures. The complex surface requires more time to observe and interpret. Furthermore, for bamboo culms with stripes, the average gaze time of bamboo treated at 80 °C is the highest (0.56 s), followed by 70 °C (0.51 s) and 60 °C (0.48 s) in succession. This could be due to the complexity of surface information increasing with an increase in stripe density correlated to temperature (Figure 4a). 

**Figure 7:**
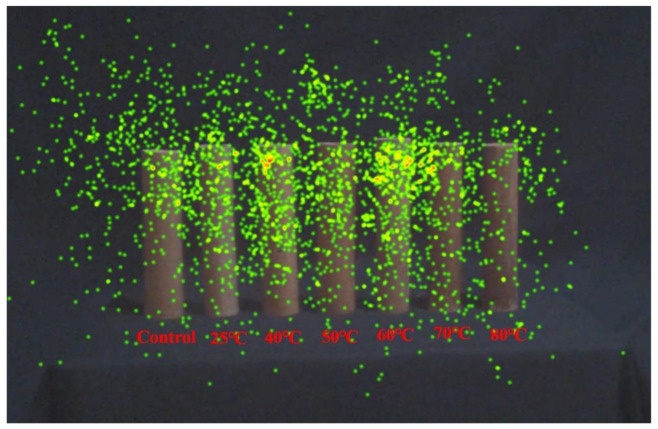
The heat map of bamboo culms in eye tracking test.

The heat map showed the overall effect of all the gaze points on the samples [53]. The visual intensity mapped here reflects the degree of preference for each treatment at each temperature. The gaze frequency and time are represented using a color gradient between green-yellow-orange-red. Green represents a lower gaze frequency and shorter gaze time, while red represents a higher gaze frequency and longer gaze time. Figure 7 intuitively shows the gaze regions and visual presentation. From the heat map, the gaze point concentration area is distributed between the 60 °C and 40 °C treatments. The control group and bamboo treated with 70 °C and 80 °C have fewer gaze points. Considering the three factors of color, gloss and texture, the light color, appropriate gloss and sparse stripes may attract the most attention of all the bamboo culms. On the contrary, dark color, high gloss, and dense, uneven stripes attract less attention. The lower lightness and uneven texture at 80 °C received very little attention. Similarly, the bamboo culm treated at 50 °C with lower lightness and higher gloss obtained less attention than the culm treated at 40 °C. 

**Figure 8:**
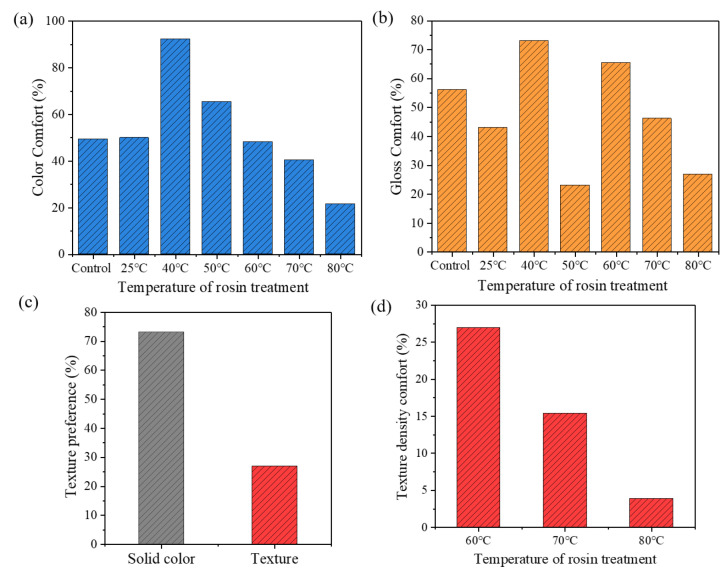
Subjective comfort evaluation results of color, gloss and texture preference of testees on bamboo culm surface. (**a**) color comfort; (**b**) gloss comfort; (**c**) texture preference; (**d**) texture density comfort.

The subjective reviews respond to the “color”, ”gloss”, and “texture” preference of testees. The results are shown in Figure 8. It shows that: (1) in terms of color comfort, the comfort preference of bamboo culm treated at 80 °C was obviously lower than that of the control group, and higher comfort recognition was obtained at 40 °C and 50 °C; (2) as regards the sense of gloss comfort, the bamboo culm treated at 25 °C, 50 °C, and 80 °C obtained the lower comfort recognition than control. Among these, the gloss at 25 °C and 80 °C were considered dull, while that at 50 °C was considered dazzling; these may be the reason why bamboo culms at 50 °C and 80 °C are less concerned than others; (3) by the texture preference, 73% of testees preferred a solid color bamboo surface, while 27% preferred texture one; this shows that the bamboo with solid color surface has a wider audience, and the textured surface is also loved by a small number of people; (4) additionally, as for texture density, the comfort recognition gradually decreased from 60 °C to 80 °C. It could be inferred that texture comfort weakened with the increase of texture density. 

From the results of subjective reviews, it could be inferred that the increased gaze time and number of bamboo culm treated at 60 °C and 70 °C may be results of its texture attracting more attention and requiring more time to observe. However, it could not infer that subjects prefer stripes from the more gaze time and numbers, because the proportion of testees who prefer stripes in subjective comfort evaluation is much less than that of pure color. Besides, the great color and gloss comfort recognition also contribute to higher attention.

## 4. Conclusions

In this study, natural resin rosin was used to modify bamboo culm at different temperatures. The visual characteristics were then analyzed quantitatively and qualitatively. At room temperature (25 °C), rosin was deposited in a granular form on the bamboo surface and increased the roughness of culm. This reduced the surface gloss but had little effect on the color of the bamboo. A proper heating treatment (40 °C and 50 °C) was conducive to the formation of a continuous rosin film, which then improved the gloss value. At these temperatures, the color of the bamboo culm also changed from green and yellow to red and blue. As temperature rose to 60 °C, the bamboo epidermis was shed. Some stripes were formed due to the shedding of the epidermal layer, and these became denser with the increase in treatment temperature. The results of the eye tracking experiment indicated that rosin modification at any temperature increased the observer’s visual interest in the bamboo culm. From the rosin-treated culms, those with stripes formed by the shedding of the epidermis increased the gaze time of bamboo culm. However, compared with textured bamboo, solid-colored surfaces get more preference. Lastly, the appropriate improvement of brightness and gloss also increased the attractiveness of the bamboo culm. Quantitative color and gloss measurements, combined with qualitative eye tracking experiments, can more comprehensively analyze the effect of rosin on the visual characteristics of the bamboo surface. Natural rosin resin could effectively improve the visual characteristics of bamboo culm, and different visual effects on bamboo culm surfaces were obtained in different temperature ranges. 

## Figures and Tables

**Table 1:** Change in color and gloss of bamboo culm after rosin treatment with different temperature.

		Color							Gloss
Bamboo Culm Sample		L*	a*	b*	ΔL*	Δa*	Δb*	ΔE*	
Control group		57.02	7.26	26.08	/	/	/	/	8.8
Rosin-treated group	25 °C	57.17	6.57	26.17	0.14	−0.69	0.09	0.71	5.6
	40 °C	56.52	7.50	26.74	−0.50	0.24	0.66	0.87	12.4
	50 °C	53.43	8.46	25.66	−3.60	0.20	−0.42	3.81	19.6
	60 °C	52.57	8.71	23.89	−4.45	1.45	−2.19	5.16	15.5
	70 °C	49.07	8.19	22.87	−7.95	0.92	−3.21	8.62	12.9
	80 °C	43.80	8.98	18.88	−13.22	0.72	−7.20	15.15	6.8

## Data Availability

Not applicable.

## References

[1] Van der Lugt P., Van den Dobbelsteen A.A.J.F., Janssen J.J.A. (2006). An environmental, economic and practical assessment of bamboo as a building material for supporting structures. Constr. Build. Mater..

[2] Liese W., Köhl M. (2015). Bamboo.

[3] Manandhar R., Kim J.H., Kim J.T. (2019). Environmental, social and economic sustainability of bamboo and bamboo-based construction materials in buildings. J. Asian Archit. Build. Eng..

[4] Miclat M.C. (2000). The visual poetry of Chinese bamboo: Some notes on traditional Chinese Xieyi painting. Humanit. Diliman.

[5] Wang X.Y. (2012). Study on aesthetic application of the original bamboo in assembled bamboo buildings. Adv. Educ. Res..

[6] Weibel D., Stricker D., Wissmath B., Mast F.W. (2010). How Socially Relevant Visual Characteristics of Avatars Influence Impression Formation. J. Media Psychol..

[7] Küller R., Ballal S., Laike T., Mikellides B., Tonello G. (2006). The impact of light and colour on psychological mood: A cross-cultural study of indoor work environments. Ergonomics.

[8] Levy B.I. (1984). Research into the psychological meaning of color. Am. J. Art Ther..

[9] Jiang Z. (2007). Bamboo and Rattan in the World.

[10] Jacobs K.W., Blandino S.E. (1992). Effects of color of paper on which the profile of mood states is printed on the psychological states it measures. Percept. Mot. Ski..

[11] Adams W.J., Kerrigan I.S., Graf E.W. (2016). Touch influences perceived gloss. Sci. Rep..

[12] Wastiels L., Schifferstein H.N., Heylighen A., Wouters I. (2012). Relating material experience to technical parameters: A case study on visual and tactile warmth perception of indoor wall materials. Build. Environ..

[13] Rosu D., Teaca C.-A., Bodirlau R., Rosu L. (2010). FTIR and color change of the modified wood as a result of artificial light irradiation. J. Photochem. Photobiol. B Biol..

[14] Temiz A., Yildiz U.C., Aydin I., Eikenes M., Alfredsen G., Çolakoglu G. (2005). Surface roughness and color characteristics of wood treated with preservatives after accelerated weathering test. Appl. Surf. Sci..

[15] Kamdem D.P., Grelier S. (2002). Surface Roughness and Color Change of Copper-Amine Treated Red Maple (*Acer rubrum*) Exposed to Artificial Ultraviolet Light. Holzforschung.

[16] Chang S.-T., Wu J.-H. (2000). Stabilizing Effect of Chromated Salt Treatment on the Green Color of Ma Bamboo (*Dendrocalamus latiflorus*). Holzforschung.

[17] Zhou T., Shi X.U., Wang Z., Sun D. (2002). Effect of heat treatment on texture and color changes in minimally processed water bamboo. J. Wuxi Univ. Light Ind..

[18] Feng Q., Huang Y., Ye C., Fei B., Yang S. (2021). Impact of hygrothermal treatment on the physical properties and chemical composition of Moso bamboo (*Phyllostachys edulis*). Holzforschung.

[19] Karlberg A.-T., Magnusson K. (1996). Rosin components identified in diapers. Contact Dermat..

[20] Shaw D.N., Sebrell L.B. (1926). The Chemical Composition of Rosin. Ind. Eng. Chem..

[21] Su Z.A., Liang Z.Q., Qin W.L., Jiang Z.R. (1980). Study on the principal chemical constituents of Chinese rosin and turpentine. Sci. Silvae Sin..

[22] Wang J.-F., Lin M.-T., Wang C.-P., Chu F.-X. (2009). Study on the synthesis, characterization, and kinetic of bulk polymerization of disproportionated rosin (β-acryloxyl ethyl) ester. J. Appl. Polym. Sci..

[23] Pathak Y.V., Dorle A.K. (1990). Rosin and rosin derivatives as hydrophobic matrix materials for controlled release of drugs. Drug Des. Deliv..

[24] Mayer M., Meuldijk J., Thoenes D. (2010). Emulsion polymerization of styrene with disproportionated rosin acid soap as emulsifier. J. Appl. Polym. Sci..

[25] Su N., Fang C., Yu Z., Zhou H., Wang X., Tang T., Zhang S., Fei B. (2021). Effects of rosin treatment on hygroscopicity, dimensional stability, and pore structure of round bamboo culm. Constr. Build. Mater..

[26] Satturwar P.M., Fulzele S.V., Dorle A.K. (2003). Biodegradation and in vivo biocompatibility of rosin: A natural film-forming polymer. AAPS PharmSciTech.

[27] Niu X., Liu Y., Song Y., Han J., Pan H. (2018). Rosin modified cellulose nanofiber as a reinforcing and co-antimicrobial agents in polylactic acid/chitosan composite film for food packaging. Carbohydr. Polym..

[28] Moustafa H., El Kissi N., Abou-Kandil A.I., Abdel-Aziz M.S., Dufresne A. (2017). PLA/PBAT Bionanocomposites with Antimicrobial Natural Rosin for Green Packaging. ACS Appl. Mater. Interfaces.

[29] Dong Y., Yan Y., Wang K., Li J., Zhang S., Xia C., Shi S.Q., Cai L. (2016). Improvement of water resistance, dimensional stability, and mechanical properties of poplar wood by rosin impregnation. Eur. J. Wood Wood Prod..

[30] Nguyen T.T.H., Li S., Li J. (2013). The combined effects of copper sulfate and rosin sizing agent treatment on some physical and mechanical properties of poplar wood. Constr. Build. Mater..

[31] Dahlen J., Nicholas D.D., Schultz T.P. (2008). Water Repellency and Dimensional Stability of Southern Pine Decking Treated with Waterborne Resin Acids. J. Wood Chem. Technol..

[32] Su N., Fang C., Zhou H., Tang T., Zhang S., Fei B. (2021). Hydrophobic treatment of bamboo with rosin. Constr. Build. Mater..

[33] Xu J.F., Zhang H.N. (2012). Modern Furniture Color Image Based on Eye Tracking. Appl. Mech. Mater..

[34] Tuch A., Kreibig S., Roth S., Bargas-Avila J., Opwis K., Wilhelm F. (2011). The Role of Visual Complexity in Affective Reactions to Webpages: Subjective, Eye Movement, and Cardiovascular Responses. IEEE Trans. Affect. Comput..

[35] Granka L.A., Joachims T., Gay G. Eye-tracking analysis of user behavior in WWW search. Proceedings of the International ACM SIGIR Conference on Research & Development in Information Retrieval.

[36] Jacob R.J.K. (1995). Eye Tracking in Advanced Interface Design. Virtual Environ. Adv. Interface Des..

[37] Ozcelik E., Karakus T., Kursun E., Cagiltay K. (2009). An eye-tracking study of how color coding affects multimedia learning. Comput. Educ..

[38] Holmqvist K., Nystrm M., Andersson R., Dewhurst R., Weijer J. (2011). Eye Tracking: A Comprehensive Guide to Methods and Measures.

[39] Kaufman A.A., Bandopadhay A., Piligian G.J. (1994). Apparatus and Method for Eye Tracking Interface. U.S. Patent.

[40] Wan Q., Wang G.G., Zhang Y.C., Song S.S., Fei B.H., Li X.H. (2018). Cognitve processing torword traditional and new Chinese style furniture: Evidence from eye-tracking technology. Wood Res..

[41] Suárez L.A.D.L.F. (2020). Subjective experience and visual attention to a historic building: A real-world eye-tracking study. Front. Arch. Res..

[42] (2014). ISO 2813:2014. Paints and Varnishes—Determination of Gloss Value at 20 Degrees, 60 Degrees and 85 Degrees.

[43] Brischke C., Welzbacher C.R., Brandt K., Rapp A.O. (2007). Quality control of thermally modified timber: Interrelationship between heat treatment intensities and CIE *L***a***b** color data on homogenized wood samples. Holzforschung.

[44] Habashi F. (2019). Witt and the Theory of Dyeing. Latest Trends Text. Fash. Des..

[45] Liu J.L., Liu X.M., Li W.G., Ma L., Shen F. (2014). Kinetics of gum rosin oxidation under 365 nm ultraviolet irradiation. Mon. Chem..

[46] Liese W. (1998). The Anatomy of Bamboo Culms.

[47] Luxmoore A.R. (1983). Appendix—Basic Principles of Optics.

[48] Fahey L.M., Nieuwoudt M.K., Harris P.J. (2017). Predicting the cell-wall compositions of *Pinus radiata* (radiata pine) wood using ATR and transmission FTIR spectroscopies. Cellulose.

[49] Yin Y., Berglund L., Salmén L. (2011). Effect of Steam Treatment on the Properties of Wood Cell Walls. Biomacromolecules.

[50] Artaki I., Ray U., Gordon H., Gervasio M. (1992). Thermal degradation of rosin during high temperature solder reflow. Thermochim. Acta.

[51] Evans P.D., Thay P.D., Schmalzl K.J. (1996). Degradation of wood surfaces during natural weathering. Effects on lignin and cellulose and on the adhesion of acrylic latex primers. Wood Sci. Technol..

[52] Ettwein F., Rohrer-Vanzo V., Langthaler G., Stern T., Moser O., Leitner R., Regenfelder K., Werner A. (2017). Consumer’s perception of high gloss furniture: Instrumental gloss measurement versus visual gloss evaluation. Eur. J. Wood Wood Prod..

[53] Tidén J., Klich M. (2011). Visualisering av Eye-Tracking Data—Heat Map Generator.

